# “There are more things in heaven and earth!” How knowledge about traditional healing affects clinical practice: interviews with conventional health personnel

**DOI:** 10.1080/22423982.2017.1398010

**Published:** 2017-11-12

**Authors:** Anette Langås-Larsen, Anita Salamonsen, Agnete Egilsdatter Kristoffersen, Torunn Hamran, Bjørg Evjen, Trine Stub

**Affiliations:** ^a^ The National Research Center in Complementary and Alternative Medicine (NAFKAM), Department of Community Medicine, Faculty of Health Sciences, UiT, the Arctic University of Norway, Tromsø, Norway; ^b^ Department of Health and Care Sciences, Centre for Care Research, Faculty of Health Sciences, UiT, the Arctic University of Norway, Tromsø, Norway; ^c^ Centre for Sami Studies, Faculty of Humanities, Social Sciences and Education, UiT, the Arctic University of Norway, Tromsø, Norway

**Keywords:** Sami, primary healthcare, traditional healing, reading, cultural sensitivity, Birgejupmi, Árbediehtu, traditional knowledge, medical pluralism

## Abstract

People with Sami and Norwegian background are frequent users of traditional folk medicine (TM). Traditional healing, such as religious prayers of healing (*reading*) and the laying on of hands, are examples of commonly used modalities. The global aim of this study is to examine whether health personnel’s knowledge, attitudes and experiences of traditional healing affect their clinical practice. Semi-structured individual interviews (n=32) and focus group interviews (n=2) were conducted among health personnel in two communities in Northern Norway. The text data was transcribed verbatim and analysed based on the criteria for content analysis. Six themes were identified. The participants had acquired their knowledge of traditional healing through their childhood, adolescence and experience as health personnel in the communities. They all expressed that they were positive to the patients’ use of traditional healing. They justified their attitudes, stating that “there are more things in heaven and earth” and they had faith in the placebo effects of traditional healing. The health personnel respected their patients’ faith and many facilitated the use of traditional healing. In some cases, they also applied traditional healing tools if the patients asked them to do so. The health personnel were positive and open-minded towards traditional healing. They considered *reading* as a tool that could help the patients to handle illness in a good way. Health personnel were willing to perform traditional healing and include traditional tools in their professional toolkit, even though these tools were not documented as evidence-based treatment. In this way they could offer their patients integrated health services which were tailored to the patients’ treatment philosophy.

## Introduction

Traditional medicine and healing have traditionally been integrated parts of many citizens’ healthcare practices in circumpolar areas. The World Health Organization distinguishes between complementary and alternative medicine (CAM) and traditional medicine [1]. CAM is the term for medical products and practices that are generally not taught in medical schools and are usually not offered in conventional (allopathic) medicine [2]. In Norway, CAM is a healthcare modality that is mainly practiced outside the public health service.

Traditional medicine is understood as

the sum total of the knowledge, skills and practices based on the theories, beliefs and experiences indigenous to different cultures, whether explicable or not, used in the maintenance of health as well as in the prevention, diagnosis, improvement or treatment of physical and mental illness [1].

Included in CAM are ancient traditions such as the laying on of hands and reading. In this paper we, therefore, understand traditional medicine as part of the CAM in Norway [3].

“Reading” is a form of traditional medicine and describes the treatment conducted by a healer when he or she reads a healing prayer for illness [4–6]. The skills are often inherited and come from natural gifts and teaching from older to younger people. In this study, the term “traditional healing” is understood as the cure of patients through supernatural forces, healing prayers or the laying on of hands [3]. The “laying on of hands” is understood as a hands-on contact with the patient that is combined with other spiritual links made between the patient and the provider [7]. Reading and the laying on of hands are the most commonly used traditional modalities in the area [8]. Other forms of traditional medicine are herbs and cupping, which some traditional healers include in their armamentarium [7,9]. The Saga of the Norwegian Kings (Heimskringla), from 1200 B.C., states that the Sami have performed traditional healing as a part of their workmanship for generations [10,11]. In pre-Christian times, the Sami healers were known as Noajde. The Noajde worked as religious and spiritual leaders with medical knowledge and healing abilities [12].

The Sami culture is strongly connected to the supernatural [13], and in this culture a healer reads healing prayers for people who suffer from illness. In addition, some healers have “warm” hands and can feel other people’s pain in their own body or by clairvoyance. Such abilities are called “gifts of grace” [4,5,9,14]. Traditional healing is mostly known in Northern Norway in regions with Sami populations in a Christian context and is regarded as an important part of the Sami cultural traditions [9,12,14,15]. Some healers (called “readers”) have knowledge of diseases and their cure [6,16,17]. They do not advertise their services and they do not ask for money, but people often give them small gifts [15]. Information about the healers’ abilities are not revealed to people who are perceived as outsiders [4,15]. In this respect, this is a tradition which has been preserved through secrecy [4,9,15]. However, this tradition has been made more available to the public through the media in recent years.

### The Sami people

The Norwegian state is founded on the territories of two people, the Norwegians and the Sami [18]. In acknowledgement of this, the Norwegian Parliament established the Sami Parliament in 1989 to “… enable the Sami people to safeguard and develop their language, culture and way of life” [19]. Today the Sami is an ethnic minority group in Norway with the status of indigenous people with their own language and cultural history [20]. According to the Sami Act §2–6 people can call themselves Sami and register in the Sami electoral register if they identify themselves as Sami and either have Sami as their domestic language or have a parent, grandparent or great-grandparent with Sami as his or her domestic language or have a mother or father who is registered in the Sami electoral register [19]. However, many people do not know that they are of Sami heritage [12]. It is, therefore, difficult to estimate how many Sami people there are in Norway today [21]. The numbers vary from 15,356 persons who are registered in the Sami electoral register [22] to 55,574 who live in central Sami areas in Norway [23].

The present study was conducted in two coastal Sami communities in Northern Norway with 4,000 inhabitants (2,000 in each community). Approximately 10% of these were Sami people. These communities are defined as Sami communities because they are included in the Sami language management area, which means that the population has the right to be addressed in their own language when contacting the public health services. In both communities, the Christian movement *Laestadianism* has had a great impact on their inhabitants, despite Sami or Norwegian heritage [24,25]. The Sami became Christians through the Laestadianism, a Christian layman’s movement that is named after the Swedish preacher Lars Levi Laestadius (1800–1861). Laestadius was of Sami descent and had thorough knowledge of the Sami language, culture and way of living [26]. The Laestadian revival was rapidly spread among the Sami people and throughout large parts of the Northern hemisphere by the Sami reindeer herders [27]. The Sami language and culture have been preserved through this movement [25–27].

### Health personnel

Previous research has shown that many Sami patients believe that health personnel are skeptical to traditional healing [28]. The Coordination Reform [29] states that there is a communication gap between the health personnel and the Sami users of health services. This is due to the health personnel’s lack of knowledge about the Sami way of understanding and acting when it comes to illness and healing. The Sami Parliament stresses the need for cultural knowledge to secure equivalent healthcare for the Sami population in Norway [30].

A study shows that 45% of the cancer patients in Northern Norway used CAM during a 5-year period. Seventy-four per cent of these used a spiritual forms of CAM, such as laying on of hands and prayers [31]. Other studies show that Sami patients who suffer from anxiety and depression seek help from traditional healers more often than Norwegian patients. This fact is being under-communicated to the health personnel according to Sexton and Soerlie [32], Sexton and Stabbursvik [33] and Kiil [34]. In her research, Kiil found that health and illness were perceived differently by psychiatric patients in a mixed ethnic population in Northern Troms (Norwegian county) thanby health personnel [34]. However, a pilot study that was conducted in a community in Nordland (Norwegian county) shows that the health personnel have great respect for traditional healing [14]. They remain impartial to their patients’ use of traditional healers [14].

### Aim and research question

Previous studies [14,35,36] have demonstrated that healthcare personnel have great respect for traditional healing, but we do not know if this respect affects their clinical practice. Thus, the global aim of this study is to examine whether health personnel’s knowledge, attitudes and experiences of traditional healing affect their clinical practice.

In addition, we will investigate if traditional healing can be understood as coping strategies for patients faced with stress and illness.

**Figure 1.**
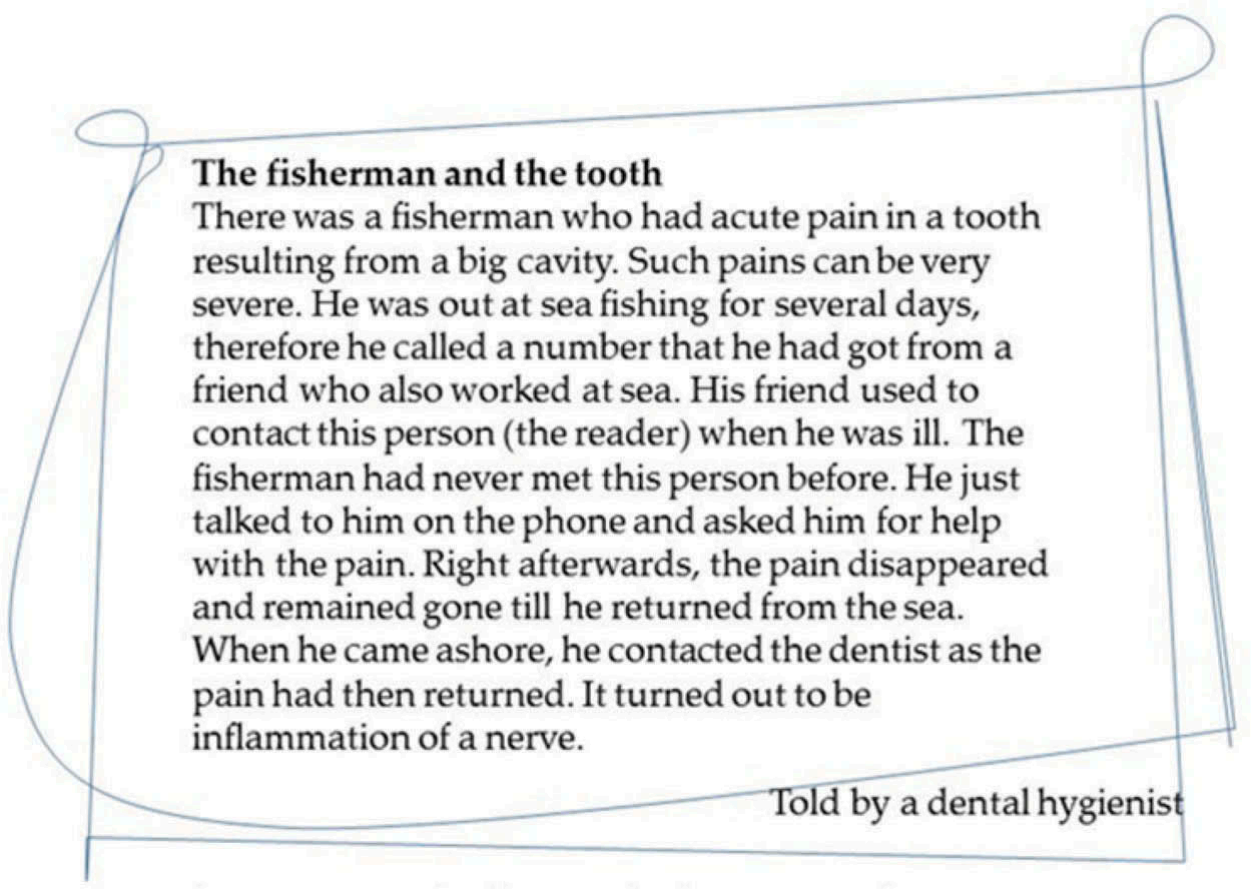
Example of a traditional healing approach.

Below is an example of a Sami healing approach (Figure 1).

## Method

### Design

This is a qualitative study consisting of 32 semi-structured individual interviews and two focus group interviews. Qualitative design is useful when examining a phenomenon of previous limited knowledge [37]. To gain knowledge about traditional medicine, it is important to understand the treatment in a philosophical and medical context [38]. The qualitative design is suitable for generating such information [39,40].

### Participants

The participants were an inter-professional group of healthcare providers who worked in primary healthcare. The demographics of the participants in this study are typical for the healthcare personnel who worked in these communities. The group consisted of four physicians, two dentists, 11 nurses, six nursing assistants, one social educator, one medical secretary, one counsellor, one dental hygienist, one physiotherapist, three paramedics (one was trained as a nurse) and two assistants. The average age was 46 years (n=32) and 23 were women and nine were men. The employment period varied from 3 weeks (intern) to 20 years. Two thirds of them had grown up in the study communities (n=21). More than half (n=18) regarded themselves as Norwegians, six as Sami, one as Kven and five as mixed (Norwegian, Sami and Kven), whereas two were of European descent. Thirteen participants had parents who spoke Sami as their native language and 12 of them had a traditional healer in their immediate family (parents or grandparents). Seven of the participants who currently speak Sami have a traditional healer in the immediate family. All participants participated in the individual interviews; of these eight participated in the focus group interviews (Table 1).

### Recruitment

**Figure 2.**
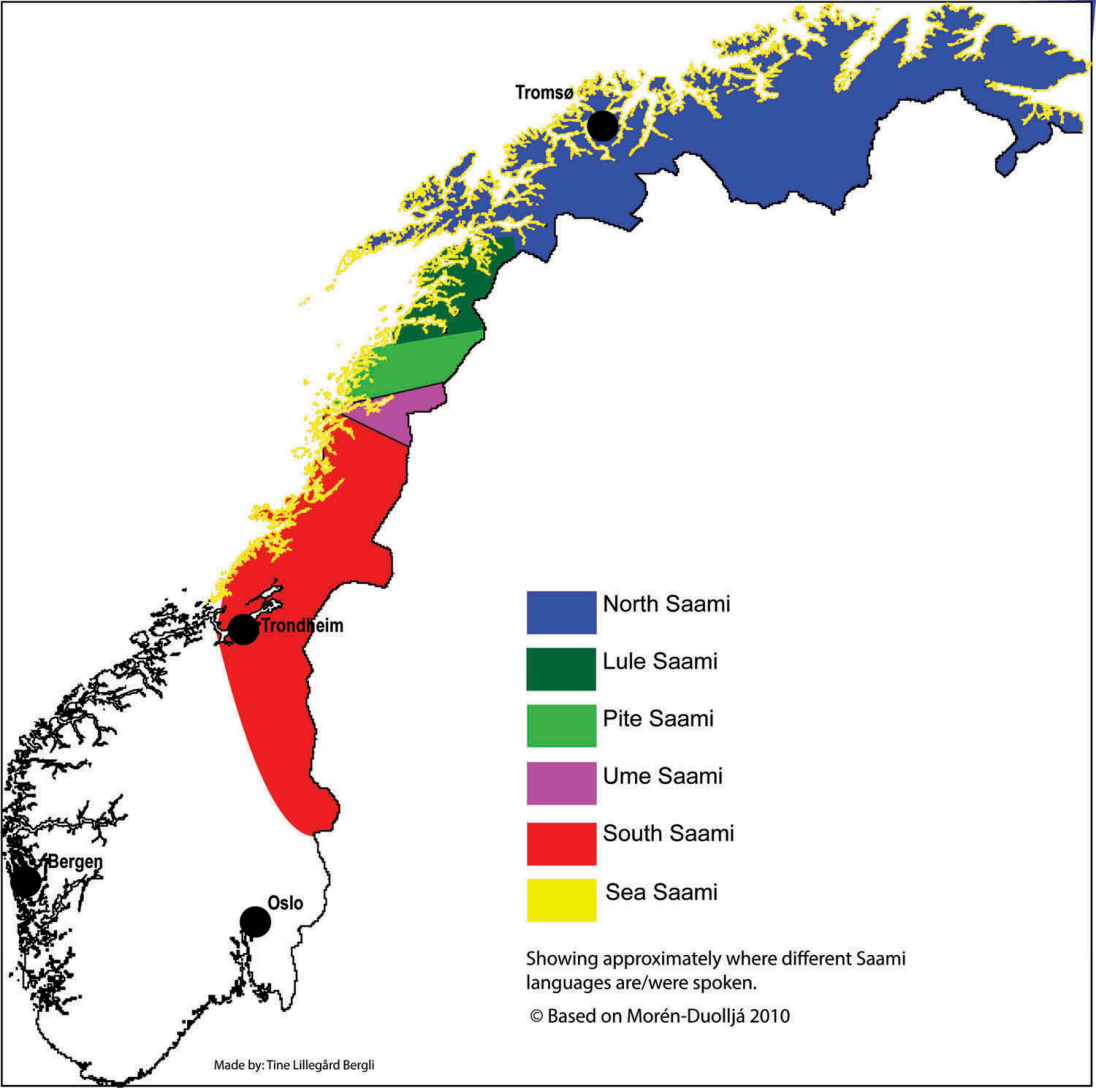
Sami settlements and where different Sami languages are spoken in Norway.

The study was conducted in close cooperation with the head of health and care services in both communities. They informed the community staff and recruited 23 participants. In addition, nine participants were recruited through recommendation from the other participants [41,42]. We wanted information from all the different healthcare professionals who worked in the healthcare service in the communities. However, after 32 interviews we did not gather any new information and decided to finish the inclusion process [43]. All interviews except two were conducted at the participants’ workplaces. Upon request from two participants, they were interviewed in their homes. The participants in the focus group were recruited by the head of health and care services in the two communities and were conducted at the workplace. Below is a map of the Sami settlements in Norway based on the map of Morén-Duolljá [44] (Figure 2).

### Individual semi-structured interviews

We chose to use individual semi-structured interviews to reveal information about the participants’ everyday practice told through personal stories and experiences [45]. Semi-structured interviews make it possible to acquire new knowledge about specific issues. The design allows the interviewer to use questions from an interview guide, but also to create questions during the interviews [37]. These interviews also open for discussing sensitive issues, such as traditional healing [37]. The interviews lasted for 30–60 minutes and were mainly conducted during the participants’ working hours. The participants who took part in the brief interviews (30 minutes) had less to say because their knowledge about the topic was limited. They originally came from other areas in Norway and Europe where traditional healing is less common. Others attended the interview unprepared, because they had received limited information about the study from their employer. Nevertheless, it was important for us to include these interviews in the study, because they provided us with a realistic picture of the variance in knowledge about traditional healing among healthcare personnel in these two communities.

The interview guide was based on a literature review, tested in a pilot study [14] and used in an interview study with healthcare providers [39,46]. The interview guide was used both in the individual and the focus group interviews. Main themes were health personnel’s experience with “reading” and their knowledge of and communication with patients and colleagues about traditional healing. The interviews resembled a normal conversation between two people. The questions were open ended, which allowed the participants to talk freely about issues of importance, according to their opinion (Table 2).

**Table 1.** Sample characteristics.

Participants	n (total = 32)
*Ethnicity*	
Norwegian	18
Sami	6
Kven	1
Mixed (Kven, Sami and Norwegian)	5
European	2
*Gender*	
Female	23
Male	9
*Age*	Average: 46 years
Nurse	55.3
Physician	38.3
Medical secretary	48.5
Paramedic	40.0
Assistant/Untrained	37.5
Nursing assistant	56
Social educator	52
*Grew up in the participating communities*	
Grew up in the community	21
Grew up outside the community	11
*Profession*	
Physician	4
Dentist	2
Nurse/Nurse specialist	11
Social educator	1
Nursing assistant	6
Medical secretary	1
Executive officer, counsellor and manager	1
Dental hygienist	1
Physiotherapist	1
Paramedic	3 (1 was a trained nurse)
Assistant/Untrained	2
*Language (experience)*	
Speak Sami	9
Language course participation	2
Speak Norwegian only	21
*Parents’ language*	
Parents who speak Sami as their domestic language	13
Finnish	1
Parents who did not speak Sami	18
*Healer in immediate family*	13
Do not have healer in immediate family	19
*Years in clinicalpractice*	
0–5 years	7
6–10 years	7
11–15 years	5
16–20 years	2
More than 20 years	11

**Table 2.** Questions asked in the interviews.

*Background*
What is your profession? For how long have you worked in the community? How old are you? Where do you live and where did you grow up? What is your ethnic identity?
*Opening questions*
Are you familiar with traditional healing and reading? What do you know about the patients’ use of reading and traditional healing?
*Relevant themes*
What are your experiences with reading and traditional healing at work? What are your experiences with reading and traditional healing in private? How did you become familiar with traditional healing? For what kinds of illness and distress do the patients seek help from a healer? How do you respond to patients who broach the issue of traditional healing and reading? Do you document the patients’ use of traditional healing and reading? (Why or why not?) In your opinion, what’s happening when someone is being read for or receives traditional healing? How does your ethnic background (Norwegian/Sami/Kven) affect your understanding of reading? Have people become more open towards reading and traditional healing while you’ve worked for the community?
*Closing questions*
Is there anything you’d like to add which we haven’t talked about? How did you experience being interviewed about traditional healing/reading?

### Focus group interviews

Focus group interviews generate different types of knowledge compared to individual interviews. Group dynamics may reveal issues and reflections of individual practice and experience that may not be disclosed in individual interviews [42,46]. One of the groups consisted of three female health workers of Norwegian (n=2) and European (n=1) descent. The other group consisted of four females and one male of Norwegian (n=2) and Sami (n=3) descent. All the focus group participants also participated in the individual interviews. The focus groups interviews were conducted after the individual interviews.

The themes discussed in the group interviews were identical to those of the individual interviews. However, the participants were encouraged to elaborate, reflect on and exemplify using their own experience. The focus groups interviews lasted less than 2 hours. The first author conducted all interviews (individual and focus groups). They were tape recorded and transcribed by the first author and a professional transcriptionist. Prior to the interviews, the participants were informed about the study both orally and in writing. All participants signed a form of consent and were informed of the possibility of withdrawal during and after the interviews.

### Data analysis

The analysis was conducted according to conventional and direct qualitative content analysis [47]. The success of content analysis depends on the coding process. In the present study the codes were defined both prior to and during the data analysis (mixed type) [37]. The text material was categorised into codes and organised according to pre-defined themes from the interview guide (“Traditional healing as coping strategies integrated in the Sami culture”, “Acknowledgement of traditional healing within the context of own practice” and “Sami patients may be misunderstood in a biomedical healthcare context”). After reading the text several times, three codes emerged from the material during the analysis (“reading”, “traditional healing from a biomedical perspective,” and “willingness to incorporate traditional tools into own clinical practice”). The text data was organised in two columns, one for text data and one for quotations. Quotations were chosen if they described a typical situation and provided a deeper understanding of the themes. The first and last authors performed the coding separately. They met and decided which codes to include in the analysis. The participants were given the opportunity to read and approve of their own interviews before publication. A native English speaker translated the quotations into English.

## Results

The results section focuses on the content of each of the six themes.

### Reading

“Reading” is a healing tradition presently known and used in areas of Sami population in Northern Norway. All participants were of the impression that “reading” was an important part of the healing tradition. However, they had not learned about traditional healing/ “reading” in professional settings. Data from our study revealed that health personnel who worked and lived in these two communities had not been informed about this tradition during their formal training or when they were employed by the community. Neither the communities nor the educational institutions informed them of the cultural context. The informants had mainly acquired their knowledge of traditional healing on their own or from their families’ experiences with “reading” when they grew up.

All participants had a positive attitude towards traditional healing and wanted to learn more about “reading”, its cultural context and the Sami health in general. The material revealed that there was a difference in how physicians and other health personnel acquired knowledge about “reading”. The physicians mainly knew about “reading” from their work with patients in clinical practice. Two recently employed physicians had no knowledge of “reading” and traditional healing. Experienced physicians (employed for more than 20 years) had limited knowledge, whereas one physician had personal experience with “reading” from his childhood. He said:

P (participant): Of course, I have heard about it. I have read about it, but I have to say, I have never tried it, directly personal … (Nils, physician).

P: Some patients have told me about reading. However, no one has told me in detail the meaning of it (Marita, physician).

One of the participants who grew up with traditional healing put it this way.

I usually say that you don’t need to know everything. Because there are more things in heaven and earth than we need to know. If “reading” helps, we don’t need to know why. She continues. “I’m not curious to know either. I just know that it helps. That’s it. That’s good enough for me” (Hanna, nursing assistant).

### Traditional healing within own practice

The health personnel did not generally speak of “reading” in their encounters with their patients if the patients did not address the theme themselves. However, if the medical treatment had no effect, one participant recommended that the patient should seek help from a healer:

P: There was violence, extreme violence, smashing and destruction. I asked the mother if she had contacted a healer for the child. I said: Do you want me to get help? Yes, she said she would like that, because it was awful for her too. So, I did (Trine, social educator).

The paramedic personnel told us that they respected patients who were Christians (Laestadians) and who believed in “reading”. In such situations, they changed their clinical behaviour.

I (interviewer): What do you need to look out for?

P: How you do things, how you choose your words, how much medication you give, because they do not want to be intoxicated. Then they (the Christians) feel dirty in a way. That was my impression (Anne, paramedic).

A nurse recalled an experience with a patient many years ago. She attempted to take a blood test. She remarked that the man had good veins:

P: “No”, he said. “Try to take a blood test, but you won’t get any blood”. “No blood?” I said. “No, you won’t get any, try and you’ll see”. OK, I thought. I had to take a sample. But I couldn’t get a drop. Nice veins. “Oh no!” I said when I couldn’t get any blood, it’s impossible, I will have to stop. “OK”, he said, “try again now, you will get blood this time”. So I started all over again, and there it was. That’s what happened. Did I not get it right the first time, what actually happened? (Sandra, nurse).

Afterwards she started thinking that the man probably possessed the gifts of grace.

Many of the participants said that they changed their clinical practice after meeting patients who used traditional healing. They facilitated patients who wanted contact with a reader. They also showed Christian patients respect and some learned the Sami language to understand their patients better.

### Applying traditional tools

Traditional healing is a practice that is not taught in medical (allopathic) schools. However, to meet the patients’ needs, some participants performed “curing”. “Curing” is a concept in traditional healing that comprises both “reading a prayer” and applying “tools” during the healing ritual [48]. Steel is a material which is often used, in the form of knives or scissors. During curing, the steel is placed where the patient hurts or the steel can be used to scare demons away. In this respect, the steel has a protective effect. A female participant who worked in psychiatry told a story:

P: Some years ago I was with an elderly woman. We went swimming, and she was so happy. She hadn’t been swimming since she was a young girl, so this was a happy moment for her. When we got her out of the water, she became worried, she became extremely worried. She thought she’d got the dead ones on her from the sea. She was really worried, so full of anxiety that she needed someone to use steel on her to protect her, get rid of it. There was a big knife there in the lavvo (Sami tent) so she (the patient) told me what to do, so I used the knife to protect her from the dead ones.

I: What did you do?

P: She wanted the sign of the cross to be made upon her, on the front and back of her body, I think it was. I had to use that knife on her back, and she wanted the sign of the cross to be made, and on the front of her body too. I did what she said. Afterwards all her worries were gone (Tove, nurse).

The participant keeps on telling the researcher that this was the most effective thing she had ever done for a patient. According to her, she saved the patient from hospitalisation and heavy medication. Sometimes paramedics in the study had found themselves in situations where they chose to perform “reading”. In this story, Erik used bible verses as tools.

P: … And then she asked me if I could read. I was in the back with her then. No, I said, I can’t. She said that she could teach me how to take away her pain. I had to write some words on a piece of paper, I think they were Bible verses, I had to read them and learn them by heart. I read them for her. She said she felt better, but I don’t know. I did it for her, because she wanted me to (Eirik, paramedic).

Another tool often used in traditional healing is prayer clothes, which are small pieces of fabric on which “Jesus lives” is written. It is often a healer who gives the patient such a cloth. According to the tradition, the cloths have healing power, and the patients wear them when they become seriously ill. Several participants told of patients who have attached prayer cloths to their clothes. One participant stated:

P: Yes, they were fastened, those safety pins, or those cloths were fastened by safety pins on those hospital shirts. So sometimes you got anxious about sending them to the laundry, worried that they could disappear at the launderette. You had to be very careful that you didn’t throw away those prayer cloths (Heidi, nurse).

The health personnel in this study facilitated the use of traditional healers for their patients. In addition, they applied traditional tools when the situation called for it. The steel was used to ease anxiety, bible verses were read to reduce pain and prayer cloths were taken care of. They took the patient’s faith seriously and were willing to include these tools in their professional toolkit, even though the practice was not described in any textbook. In order to meet the patients’ healthcare need, one nurse commented:

P: Sometimes you have to do things that are not written in the textbook (Tove, nurse).

### Sami patients in a biomedical healthcare context

Some participants had learned the Sami language to better understand their patients. In their opinion, speaking Sami improved their level of understanding these patients. Even though many patients could speak Norwegian, they switched to Sami when they got dementia. A nursing assistant told about a patient who had asked for water (in Sami) for an entire day and the personnel did not understand her request.

P: … she lay (in bed) screaming for water. And if you don’t know Sami, you couldn’t understand what she was saying. Yes, I feel I get better contact with them (Inger, nursing assistant).

The Sami culture is strongly connected to the supernatural [13]. Many grew up surrounded by people who had visions or contacts “on the other side”. Some were, for example, able to hear things that others did not hear or they were able to receive warnings from animals or dreams. Several participants claimed that people with such abilities might be regarded as psychotic in a biomedical context.

P: … Oh I think that a lot of the time they will try to drug it away. Something that’s natural for us, but abnormal in the Norwegian world. This is an area in which health personnel have a lot to learn, because it isn’t abnormal, it’s completely natural (Anna, nursing assistant).

One of the nurses elaborated:

P: At least I’m very aware of that, as I experienced when I worked in Bodø. Patients were often submitted to acute psychiatric care. They were often misunderstood, regarded as more diseased than they actually were. What was interpreted as disease was often related to their faith, including contact with the deceased and the issue of dreaming. Yes, I think, at least for me, it was important to have that knowledge from the Sami area (Tove, nurse).

Most of the health workers in this study were not Sami. One of the physicians reflected on this and said:

P: … I don’t say that, good heavens, I guess we can all make mistakes. I probably have many times. I think that the health service and most health workers (in practice) are mainly concerned with offering a good standard of healthcare to people, regardless of cultural background and nationality and so on. But it’s obvious that it’s not always easy to acquire knowledge of what is typical for the Sami people and their understanding of illness (Hans, physician).

The participants in our study demonstrated willingness to understand the Sami patients and they had knowledge about the community culture context from their childhood and after having worked in the community for many years.

Nergård [12] suggests in his research that many Sami people have an “extended” and “animistic” worldview. This means that the Sami people experience nature as “alive” and elements from (the) nature may influence their health in a positive or negative way. It is, therefore, important that health personnel have knowledge about the Sami culture and belief system when assessing the patients’ symptoms and complaints.

### Traditional healing as coping strategies

The participants claimed that “reading” was part of the patient’s faith, which contributed to mobilising hope and relief in situations of illness. A nurse expressed:

P: It gives hope, you know, if you’re ill and feel miserable and think it may help. I believe that it means a lot to the patients. When they are feeling hurt and in need of comfort, it helps to have someone to read and thereby provide relief. I know it works from personal experience (Irene, nurse).

Another nurse also talked about reading as “comfort” during illness. She said:

P: When you experience difficulties, you have to find comfort in something (Sofie, nurse).

Some understood “reading” as an extra insurance in life. A paramedic explained:

P: Yes, I think it’s insurance … because they have an alternative that’s closer to nature than the medication. The medicine that we give also works, that’s true, but – you don’t get worse from the reading (Eirik, paramedic).

According to our informants, the patients used “reading” because it gave hope and comfort in periods of illness. “Reading” may, therefore, be understood as a tool that makes it easier for the patients to cope with illness. Moreover, “reading” is a coping strategy that is integrated into the Sami and the Northern Norwegian culture.

### Traditional healing from a biomedical point of view

Placebo is inactive treatments that give positive physiological, behavioural, emotional and cognitive effects. These effects often result from the “expectation of effect”. If a person experiences this inactive treatment as positive, it is called a placebo effect. The placebo effect can emerge from different forms of treatment, such as using ineffective pills (sugar pills). In some cases, a placebo can work even when the test subject knows that the treatment does not work [49]. Several of our informants claimed that “reading” had such placebo effects.

P: I think this may be a little more psychology in a way, when it comes to traditional healing and things like that. When you ask someone to read for you, you must probably have strong faith in it yourself. I’m convinced that it works if you believe it will (Frank, physiotherapist).

One dentist told a story of a terminally ill woman who wanted to live past her son’s 20th birthday.

P: But she fought all the way through to the day he turned 20, and the day after she died. So that the power of thoughts is great, that I believe. Another physician explained: It has to do with faith, the mental, it can be the placebo effect (Nils, physician).

Several participants were present in a focus group interview in which placebo was discussed.

P: Yes, you can only look at the research in medicine. They have an effect, especially the studies of antidepressants where some get antidepressants and others get sugar pills. There’s in fact improvement in those who have taken sugar pills. And then there’s the placebo effect, and that will of course always be there (Tove, nurse).

The physicians and the physiotherapist in our material understood *reading* as a placebo effect where the patients mobilised their own power. The nurses and other health personnel were of the opinion that faith, the patients’ experiences and the need for comfort, including an extra insurance in life regarding illness, were important resources for the patients. Especially for patients connected to the Sami culture or who lived in areas that have historically been Sami.

## Discussion

This study has provided increased knowledge of how health personnel handle and understand “reading” as a form of traditional healing in two communities in Northern Norway. Our findings show that the health personnel are open-minded and positive to the patients’ use of “reading”. Their explanation is that the effect mechanism cannot always be scientifically explained, such as is the case with readings. Their knowledge of traditional healing affects their clinical practice in that they facilitate “reading”. Some of the participants performed “reading” and “curing” on patients who wanted this. They were willing to use traditional tools to meet the patients’ needs. According to the participants in this study, “reading” was a coping strategy that patients and relatives employed to handle stress and illness. Moreover, when patients’ culture and faith are taken seriously, the results may be a more balanced relationship between the patient and the healthcare personnel, which is a cornerstone in patient-centred healthcare.

In the Sami tradition, the term *birgejupmi* describes skills that are employed to “save oneself” [13]. Guttorm and Porsanger [13] relate the Sami term *Árbediedu* to the collective knowledge that the Sami people have acquired through hundreds of years to survive. This knowledge has been passed down from generation to generation. In pre-Christian times, the Sami healers were known as Noajde. The Noajde worked as religious and spiritual leaders with medical knowledge and healing abilities [12]. Some of the elements of the Noajde tradition were kept and transformed into a Christian context where the use of the ancient Sami gods (such as *Beiwe*, *Madder-akka* and *Zarakka* [50]) were replaced by the Christian God [12,15]. The use of steel and elements from nature can be traced back to this ancient Sami healing tradition, which is still being used along with elements from Christian liturgy, such as prayers like The Lord’s Prayer [12].

Such continuity connects the term *árbediehtu* to previous, present as well as future knowledge. *Reading* is a tool *– birgejupmi –* that the Sami people have employed against illness for generations. *Árbediedu* describes the traditional knowledge on which “reading” is built. This is in line with other studies that related the term *árbediehtu* to coping strategies [51,52]. In our material, we found that traditional healing was used on an individual level when patients employed “reading” to mobilise their own power to handle illness.

In Norway, the inhabitants may choose from multiple healing modalities outside the conventional healthcare. However, results from this study demonstrate that patients also want to use traditional healing when inside the conventional healthcare system. In situations like this it is important that healthcare personnel bridge the gap between the culture of conventional medicine and the patients’ belief system [53]. This can be achieved by increased understanding, cultural awareness, open-mindedness and further education [53,54]. The participants in our study demonstrated a willingness to understand their patients and to facilitate healing practices.

### Implications for practice and further research

“Reading” or the use of other traditional forms of healing is seldom documented in the patients’ medical records. According to the recommendations from the University Hospital of Northern Norway, the patients’ use of “reading” should be documented in the medical records [55]. There are vast cultural differences within the Sami culture and various Sami groups have different cultural expressions and healing traditions. Different healing traditions within the Sami culture should be investigated using qualitative designs. The focus should be on similarities and differences in traditional medicine between various Sami populations (North Sámi, Lule Sámi, Pite Sámi, Ume Sámi and South Sámi). A large-scale survey among traditional healers and their patients should be conducted, with the scope to investigate who the users in the different Sami populations are.

### Methodological aspects/limitations

One of the strengths of this study is that the first author is a Sami and has grown up in one of the communities in which the study was conducted. As she was familiar with the Sami culture, she gained access to information about traditional healing which would otherwise probably have been inaccessible. Research has shown that ethnic affiliation and knowledge of traditional healing are important to gain access and build trust with indigenous people when examining this theme [4,14]. On the other hand, this might also be a weakness. As a researcher of your own culture it may be difficult to observe the obvious in that culture [56]. Still we think that the total experiences of the authors contribute to analytical depth. The second author has her roots in the other community where the study took place. She has a sociological background. The third author is a healer and the last author is an alternative therapist (acupuncturist and homeopath). The professional background of the research group has contributed to analytical depth and distance to the data. The heads of healthcare services in the two communities were informed about the research project and permissions were granted to conduct the interviews during work hours. The timing of the interviews was a possible limitation, because some participants had to cut them short because of work obligations.

## Conclusion

Results from the present study demonstrate that the health personnel are open-minded and positive towards “reading”. The health personnel acquired knowledge and experience of “reading” through their clinical practice. They facilitated the patients’ use of this healing practice and some performed reading and curing to meet the patients’ needs. According to the study participants, reading made it easier for the patients to cope with illness and stress. In this manner they could offer their patients integrated health services which were tailored to the patients’ treatment philosophy.
